# Pigmented mammary Paget disease: a diagnostic challenge^☆^

**DOI:** 10.1016/j.abd.2024.03.007

**Published:** 2024-10-31

**Authors:** Esranur Ünal, Bengü Nisa Akay, Gökçen Gündoğan

**Affiliations:** a Department of Dermatology, Kayseri City Education and Research Hospital, Kayseri, Turkey; b Department of Dermatology, Medicine Faculty, Ankara University, Ankara, Turkey; c Department of Pathology, Kayseri City Education and Research Hospital, Kayseri, Turkey

Dear Editor,

Mammary Paget disease is a rare type of adenocarcinoma. It is closely associated with breast cancer.^1^ Pigmented mammary Paget Disease (PMPD) is an uncommon variant of Paget disease that can mimic melanoma clinically, dermoscopically, and histopathologically due to the presence of melanin pigment.^2^ Here, we present a case of PMPD with dermoscopic melanoma and Bowen's Disease (BD)-like findings, in which histopathological examination made a clear distinction.

A 45-year-old female was admitted to our outpatient clinic with a slowly expanding, asymptomatic, erythematous skin lesion with some pigmented areas on the left nipple that had been present for one year. Upon dermatological examination, a 3 × 3 cm brown-pink plaque was found on the left areola, and the nipple was flattened. The plaque exhibited centrifugal growth, asymmetry, irregular borders, and scattered scaling (Fig. 1A). Dermoscopy revealed a chaotic lesion, with numerous clues for melanoma, including a pink, white structureless area, central white lines, and segmental brown radial lines. There were also brown-gray dots arranged in lines and dotted vessels in lines in some areas, adherent fabric fibers, and superficial erosions which are characteristic of Bowen's disease (Fig. 1B‒D). Although the clinical and dermoscopic findings were inconclusive, they were suggestive of pigmented Paget disease, melanoma, and pigmented BD. The histopathological examination of the areolar incisional biopsy revealed a glandular epithelium with large pale cytoplasm, large nuclei, and prominent nucleoli that were arranged both singly and in clusters in the epidermis. Immunohistochemically, tumor cells were pan Cytokeratin (CK) and CK 7 positive, Estrogen Receptor (ER), and mucin negative (Fig. 2A‒C). Histopathology was consistent with Paget's disease based on these findings. The nipple skin biopsy of the patient, who underwent breast-conserving surgery by the general surgeon, was compatible with Paget's disease, and underlying high-grade ductal carcinoma in situ was detected.

**Figure 1:**
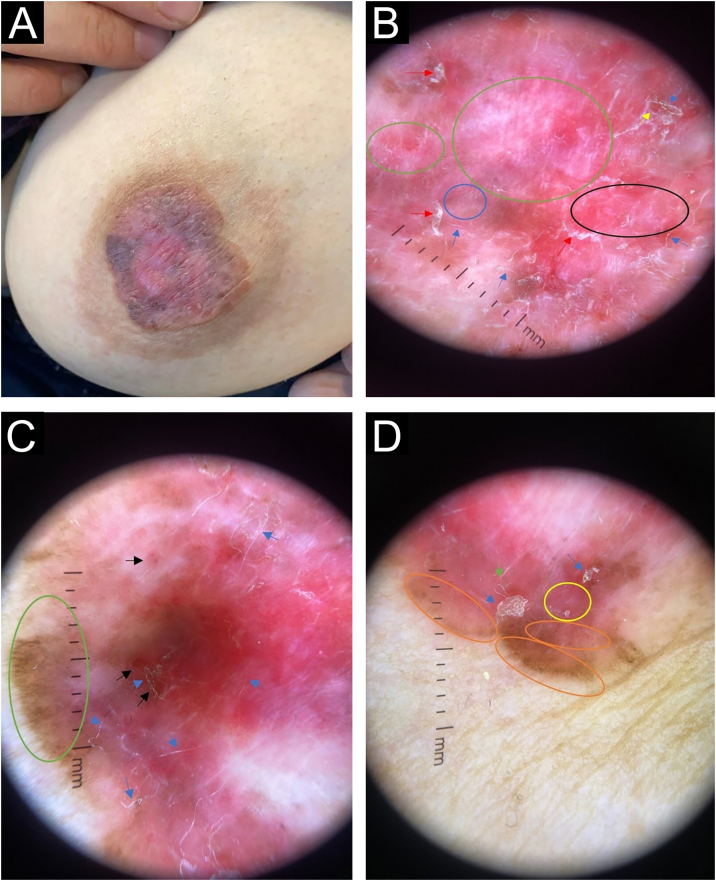
(A) Brown-pink plaque on the left areola measuring 3 × 3 cm in size, with irregular borders, and flattened nipple. (B) Red arrow: scale, blue arrow: adherent fabric fibers, yellow arrow: superficial erosions, green ring: shiny white streaks, blue ring: irregular dotted vessel, black ring: pink, white structureless area. Dermlite DL4- ×10 magnification - polarized mode. (C) Blue arrow: adherent fabric fibers, black arrow: superficial erosions, orange arrow: scale, green ring: segmental brown radial lines and brown-gray dots. Dermlite DL4- ×10 magnification - polarized mode. (D) Blue arrow: scale, green arrow: adherent fabric fibers, yellow ring: dotted vessels, orange ring: segmental radial lines and brown-gray dots. Dermlite DL4- ×10 magnification - polarized mode.

**Figure 2:**
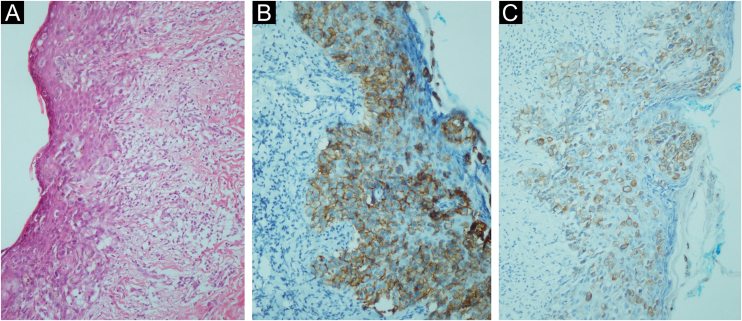
(A) Malignant epithelial cells dispersed as single or clustered cells in the epidermis (Hematoxylin & eosin, ×200). (B) Membrane staining of malignant epithelial cells in the epidermis with C-ERB-B2 (×200). (C) Cytoplasmic staining of malignant epithelial cells with CK7 (×200).

Paget disease of the nipple is a rare type of intraepidermal adenocarcinoma. It accounts for only 1%‒4% of all breast malignancies.^1^, ^3^ Dermoscopically, the most common findings of non-pigmented mammary Paget disease include white scales, pink structureless areas, dotted vessels, erosion/ulceration, and white shiny lines. On the other hand, the typical findings of PMPD are gray granules and dots, pink structureless areas, and white lines.^4^

The differential diagnosis of PMPD encompasses a large spectrum of conditions from melanoma to BD and lentigo/melanosis of the nipple and areola.^4^ Segmental brown radial lines, pink-brown structureless areas, scaling, clustered or linearly arranged brown dots, and dotted and coiled vessels can also be observed in pigmented BD. BD of the nipple is an extremely rare condition that resembles nipple melanoma; however, the dermoscopic characteristics have not been fully described in the literature.^4^, ^5^ In the medical literature, fewer than 20 cases of nipple melanoma have been published. Clinical, dermoscopic, and even confocal microscopy findings cannot reliably distinguish PMPD from melanoma.^4^

In our case, dermoscopic features are shared by both melanoma and BD, dermoscopic differentiation was not possible. In conclusion, this case once again demonstrated how difficult it can be to make a dermoscopic diagnosis of PMPD and that melanoma, Paget disease, and BD can manifest similar clinical findings or similar macroscopic findings. Therefore, in suspected cases, a comprehensive approach that includes histopathological and immunohistochemical examinations, in addition to dermoscopy, is necessary for an accurate diagnosis.

## Authors’ contributions

Esranur Ünal: The study concept and design, writing of the manuscript, critical review of the literature (The physician who examines the patient, takes the biopsy, takes dermoscopic photographs, writing of the manuscript).

Bengü Nisa Akay: Critical review of important intellectual content, final approval of the final version of the manuscript (The physician who gives advice in writing the case and evaluates dermoscopic findings).

Gökçen Gündoğan: Data collection, analysis and interpretation (Performing histopathological examination).

## Financial support

Intramural funding.

## Conflicts of interest

None declared.
